# Fabrication of modern lithium ion batteries by 3D inkjet printing: opportunities and challenges

**DOI:** 10.1016/j.heliyon.2022.e12623

**Published:** 2022-12-27

**Authors:** Kinga Sztymela, Marguerite Bienia, Fabrice Rossignol, Sophie Mailley, Steffen Ziesche, Jobin Varghese, Manuella Cerbelaud

**Affiliations:** a Univ. Limoges, CNRS, ENSCI, SPCTS, UMR 7315, IRCER, 12, rue Atlantis, 87068 Limoges Cedex, France; b CEA, LITEN, 17 rue des Martyrs, 38054 Grenoble Cedex 9, France; c Fraunhofer IKTS, Winterbergstraße 28, 01277 Dresden, Germany

**Keywords:** Inkjet printing, Lithium-ion battery, Electrodes, Printability, Three-dimensional structures

## Abstract

Inkjet printing (IJP) is a prospective additive manufacturing technology that enables the rapid and precise deposition of thin films or patterns. It offers numerous advantages over other thin-film manufacturing processes, including cost-effectiveness, ease of use, reduced waste material, and scalability. The key advantage of this technique is the ability of the fabrication of complex patterns with very high precision. The IJP gives the possibility of building three-dimensional (3D) structures on the microscale, which is beneficial for modern Li-Ion batteries (LIBs) and All-Solid-State Li-Ion Batteries (ASSLIBs). In contrast to typical laminated composite electrodes manufactured by tape casting and calendaring, 3D electrode design allows the electrolyte to penetrate through the electrode volume, increasing the surface-to-volume ratio and reducing ion diffusion paths. Thus, 3D electrodes/electrolyte structures are one of the most promising strategies for producing next-generation lithium-ion batteries with enhanced electrochemical performance. Although in the literature review, the IJP is frequently reported as a future perspective for the fabrication of 3D electrodes/electrolytes structures for LIBs, only a few works focus on this subject. In this review, we summarize the previous studies devoted to the topic and discuss different bottlenecks and challenges limiting further development.

## Graphical abstract

**Figure:**
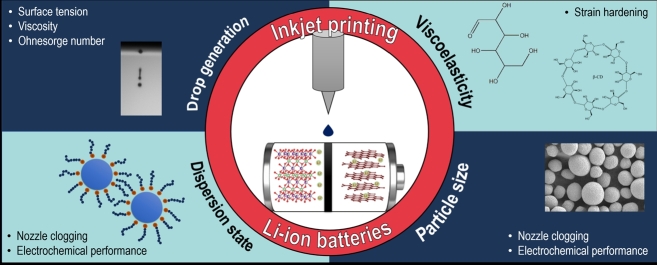

## 1 Introduction

Lithium-ion batteries (LIBs) are among the most widely used energy storage devices in the global market. Every day, they power the lives of millions of people, from portable electronics to hybrids and electric vehicles [1]. As a result, they have attracted the interest of both academia and industry. Although research on LIBs has brought remarkable results, many aspects can still be developed to improve their performance. Three-dimensional (3D) electrode structures and innovative materials development are among the most promising strategies for producing modern LIBs [2], [3].

The trade-off between energy density and power density is the main issue in typical laminated composite electrodes manufactured by a tape casting process. The energy density may be raised by increasing the amount of active material, thereby building a thicker electrode. However, this limits ion and electron mobility, leading to poor power performance [4]. As an alternative, a three-dimensional (3D) electrode design allows the electrolyte to penetrate through the electrode volume, increasing the surface-to-volume ratio and reducing ion diffusion paths. Moreover, the lithiation stress may be significantly reduced due to the free surface, which enables the use of high-capacity materials with considerable volumetric variations during the electrochemical cycles of the battery [5]. Therefore, one of the most promising strategies for producing next-generation LIBs with enhanced electrochemical performance is to manufacture three-dimensional electrodes/electrolyte structures [6].

3D printed batteries are a part of futuristic technology in emerging electronic applications. Various additive manufacturing (AM) processes can be used to create 3D battery architectures: stereolithography (SLA) [7], [8], fused filament fabrication (FFF) [9], [10], direct ink writing (DIW) [11], [12], aerosol jet printing [13], [14], and inkjet printing [15]. Among them, inkjet printing (IJP), as a prospective additive manufacturing technique that enables the rapid and precise deposition of thin films [16], [17] or 3D patterns [18], [19], is considered a promising technology for the fabrication of LIBs. The IJP process may build high-resolution multi-material 3D microstructures with a broad range of materials [20], [21]. The variety of materials that can be used makes it particularly interesting for printing complex suspensions such as electrode inks containing binders. Due to the cohesion being achieved by these binders and the evaporation of the solvent taking place between the deposition of subsequent layers, the printed product is fully functional as soon as it leaves the printing system, maintaining the shape. Post-processing steps, such as calendaring, can sometimes be done to adjust the porosity, although they are not always necessary.

Moreover, the development of All-Solid-State Li-Ion batteries (ASSLIBs) can be foreseen with this process. ASSLIBs are made up of a solid electrolyte (SE), not only eliminating the risk of explosion but also offering higher volumetric energy density than the lithium-ion batteries currently available on the market [22]. Furthermore, as the IJP gives the possibility of multi-material printing, the cell could be fabricated in one step, facilitating production. The IJP brings many advantages over other fabrication methods, such as cost-effectiveness, ease of use, minimal wasted material, and scalability. However, the major challenge is the development of a suitable ink [23], [24]. Specific requirements for the printable slurry are covered in this paper's subsequent section.

**Figure 1:**
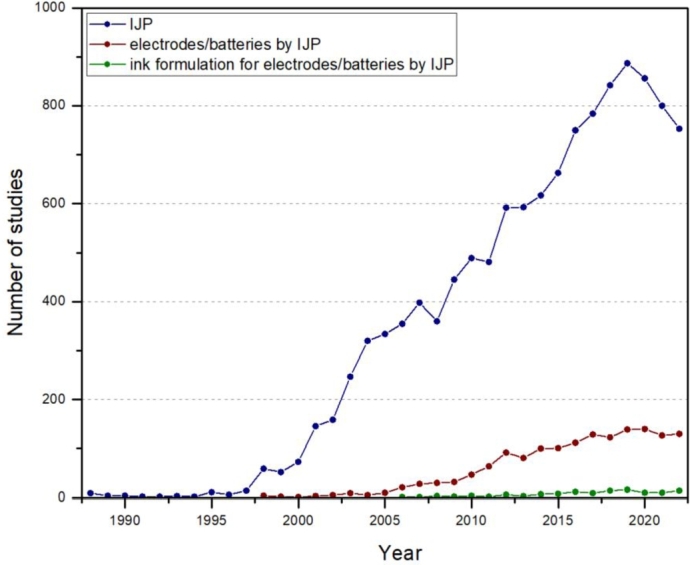
Number of studies devoted to: inkjet printing, electrodes/batteries fabricated by inkjet printing, and ink formulation for electrodes/batteries fabricated by inkjet printing over the course of years. Analysis was performed using the Scopus database (Elsevier).

3D printing techniques are expected to significantly develop in the field of batteries [25]. The IJP was highlighted as a promising technology for the fabrication of 3D designs [26], [27] and thin films [28] for LIBs. However, the understanding of drop generation and ejection processes must be improved [29]. Although IJP offers many advantages, only a few articles studied the application of this method for LIBs [16], [30]. Nevertheless, they exclusively focused on the deposition of thin films, and as far as we know, no previous research has demonstrated the 3D electrode/electrolytes structures performed by the IJP. The question that then naturally arises is, what are the potential issues limiting the progress in this field? The plot in Fig. 1 demonstrates that, since the creation of the IJP, the number of studies devoted to this technology have been continuously and rapidly increasing up to the present. At the beginning of the 21^st^ century, the IJP aroused interest in batteries, which started growing and then reached a plateau. The third green line represents studies on the inkjet printed batteries/electrodes focusing on ink formulation/rheology. There is no doubt that there is a research gap in this area. The most likely limiting factor is a lack of understanding of the relationship between ink formulation and processing.

**Figure 2:**
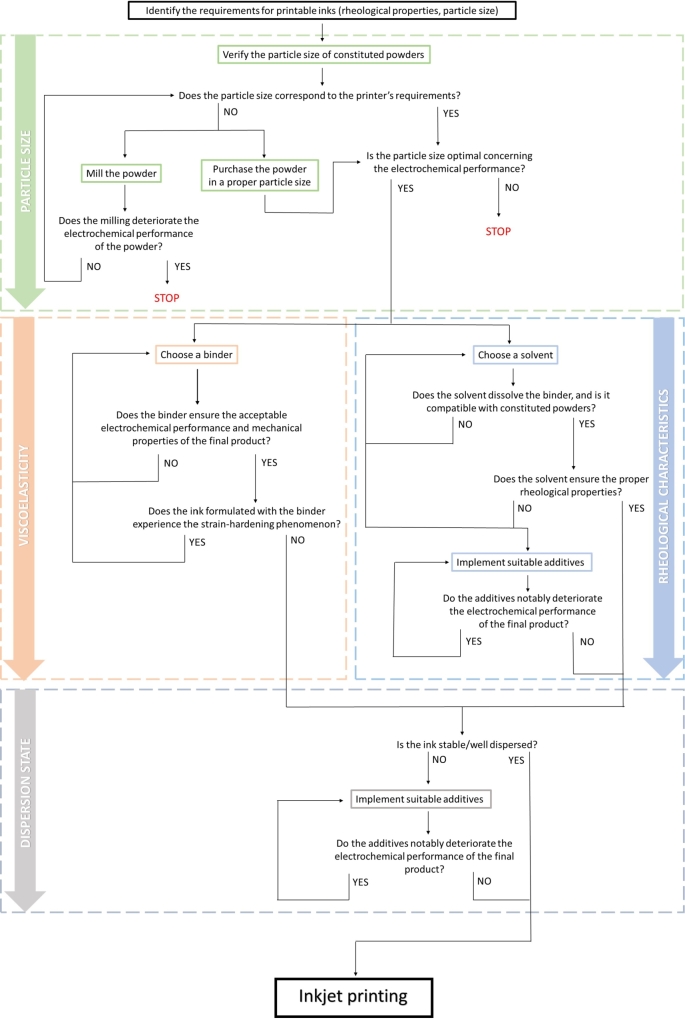
Detailed flowchart of the fabrication process of electrodes ink.

The subsequent sections point out four main issues related to ink formulation and consequently printability: particle size, viscoelasticity, rheological characteristics, and dispersion state. The flowchart in Fig. 2 illustrates the step-by-step process of ink formulation. Following these steps should aid in achieving both printability and appropriate electrochemical characteristics for printed electrodes. This review goes through these aspects in depth, highlighting various bottlenecks and challenges, with a focus on the application of the IJP method for the fabrication of LIBs. Finally, previous studies on the IJP for LIBs are summarized and discussed.

## 2 Inkjet printing technology

### 2.1 Inkjet printing principle

Inkjet printing is an advantageous and advanced printing process that enables the deposition of functional inks. Objects are constructed directly from CAD files by the placement of picoliter droplets from a liquid onto substrate [31]. Based on the mechanism of drop generation, inkjet printing technologies are divided into two categories: continuous inkjet (CIJ) and drop-on-demand (DOD) printing.

**Figure 3:**
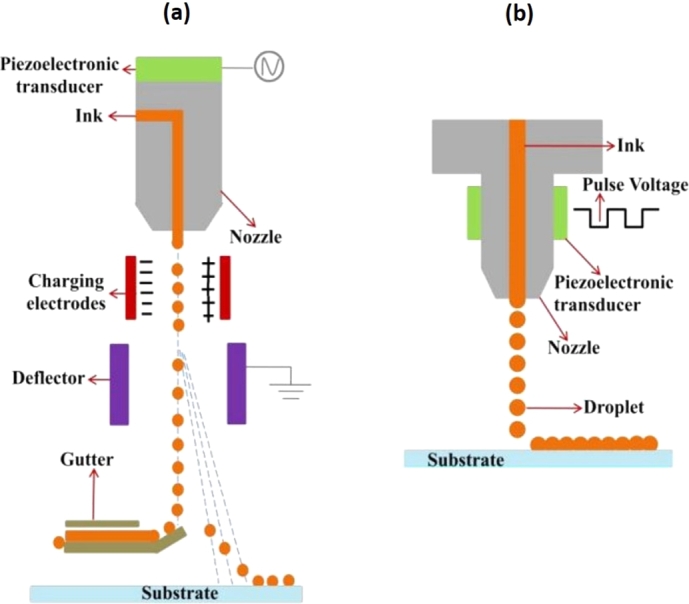
Schematic representation of inkjet printing methods: a) continuous inkjet printing, b) drop-on-demand inkjet printing. Reproduced from [34] with permission from Royal Society of Chemistry.

In CIJ printing, fluid is supplied to the nozzles via a pressure pump, and a continuous stream of drops is ejected with a piezoelectric crystal. After leaving the nozzle, the ink droplets pass through an electrostatic field and are selectively charged. Subsequently, the deflection plate deviates uncharged drops into a gutter for re-use, and uncharged drops are directed to the substrate to create a pattern (Fig. 3a). The CIJ method allows for printing at high speed, which is very useful in the industrial field. For single-line print, the speed can reach around 500 m/min [32]. Furthermore, since the droplets are generated continuously, the problem of nozzle clogging is not an issue. However, only inks that can be electrically charged may be used in this process. Another drawback is the relatively high cost of the printer [33].

In DOD inkjet printing, drops are ejected from the nozzle only when required via a pressure pulse (Fig. 3b). Various techniques may create this pressure pulse, and based on that, DOD printers are further categorized. The most common methods include thermal, piezoelectric, acoustic, and electrostatic systems. The detailed description can be found elsewhere [35], and in this review, special attention is given to piezoelectric DOD inkjet printing. The piezoelectric DOD system allows for the deposition of a variety of inks. It is considered the preferred method for most applications in printing functional materials [36]. In this technology, a pressure pulse is created mechanically by distortion of a piezo-crystal when an electric field is applied. This pulse causes the generation of droplets, which have volumes typically in the range of 1–1000 pL, yielding a high resolution of printed patterns [37], [38]. Since the ejection is executed by piezo-ceramic distortion, there is no restriction related to the thermal and electrical properties of inks. Moreover, the drop generation process can be relatively easily controlled by modifying the actuation pulse [39], [40]. A DOD inkjet printer, for instance, can print at a speed of 500 mm/s with a resolution of 5 μm × 5 μm [41].

### 2.2 Ink formulation

In order to successfully deposit the desired pattern, a stable suspension with defined properties specified for the printer must be formulated. The rheology of the ink is an essential aspect to consider since it determines whether a drop can be ejected, and how the fluid behaves once it exits the nozzle. Wolfgang von Ohnesorge demonstrated the significance of viscosity, surface tension, and fluid inertia in predicting the flow's behavior [42]. Taking into account the Reynolds (*Re*) and the Weber (*We*) numbers, he introduced a new dimensionless group – the Ohnesorge number (*Oh*), which defines the boundaries of the various operating regimes for the problem of jet breakup:

(1) $$Re=\frac{\upsilon \rho a}{\eta },$$

(2) $$We=\frac{\upsilon^{2}\rho a}{\gamma },$$

(3) $$Oh=\frac{\sqrt{We}}{Re},$$

where *υ*, *ρ* and *a* are the velocity, density and characteristic length respectively, *η* is the dynamic viscosity and *γ* is the surface tension.

Fromm [43] identified the variable Z=1/Oh as a simple method for estimating the printability of ink, assuming that a stable drop is generated when Z>2. Reis and Derby developed this concept via numerical simulations [44] and suggested the range 1<Z<10 for printable ink, which is commonly recognized in most commercial DOD printing systems [45]. However, wider ranges of printability were also reported [46], implying that this criterion is not pertinent.

**Figure 4:**
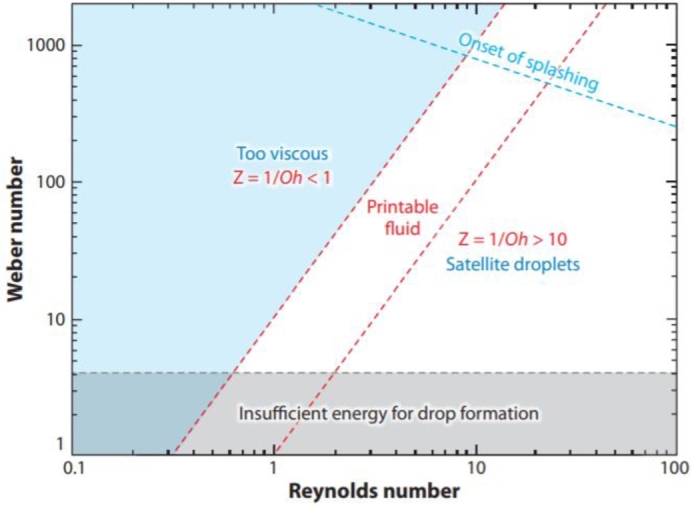
A parameter space for successful DOD printing defined by axes of Reynolds and Weber numbers. Reproduced from [39] with permission from Annual Reviews Inc.

In general, when *Z* is too low, the viscous forces prevent the drop generation, whereas, at high values, satellite droplet formation occurs. There are, however, other aspects to consider while ejecting a drop. Duineveld et al. [47] claimed that there is a minimum velocity that yields enough energy to overcome the surface tension forces of a liquid to form a drop, and accordingly, printing is possible when the *We* number is greater than 4. The impact of the drop on the substrate is another important factor to consider. A single isolated droplet should be placed without secondary droplets around. Stowe and Hadfield [48] combined *We* and *Re* values and showed that when ${\text{We}}^{1/2}{\text{Re}}^{1/4}$ is larger than 50, splashing occurs. The zone within this parameter space, where stable drops without satellites can be deposited on a substrate without splashing, is represented in Fig. 4.

However, the Ohnesorge number is unambiguously determined for simple Newtonian fluids, for which liquid properties do not depend on flow conditions. The great majority of functional inks used for the fabrication of LIBs display non-Newtonian properties. Shear-thinning behavior occurs in most concentrated suspensions [49], and with the addition of a polymeric binder, viscoelasticity may be observed, changing the drop generation characteristics [50]. The flow behavior of inks is influenced by various factors, such as concentration of solid fraction, particle size and shape, kind of added dispersing agent or lack thereof, type of used solvent, architecture, molecular weight, and concentration of the binder [51].

**Figure 5:**
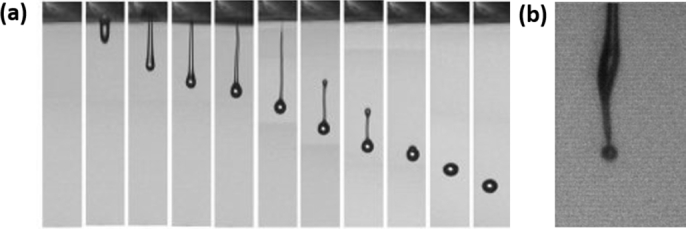
a) Ideal scenario of the droplet ejection process. Reproduced from [33] with permission from the Royal Society of Chemistry. b) Image showing the behavior of a viscoelastic ink (PVA, 25 wt% in water) after exiting the nozzle. Reproduced from [53] with permission from Elsevier.

Since DOD printing is a precise deposition method, the objective is to optimize the process parameters so that a single drop traveling straight to the substrate is formed (Fig. 5a). In an ideal scenario of the ejection process, the fluid flows out from the nozzle, forming the main drop with an attached filament. After the filament breaks up, the tail end becomes rounded and speeds up, merging with the main drop into single droplets [52].

Bienia et al. [53] demonstrated that despite the similar properties, the drop generation behavior is different for every ink, reflecting the potential impact of their particulate nature. Furthermore, although complex inks are sometimes adapted in terms of viscosity, surface tension, and stability, the ejection is still unsuccessful. For instance, the viscoelastic effect may cause the loss of axisymmetry during the ejection (Fig. 5b), resulting in poor jetting quality.

A universal window that can be used to forecast fluid behavior during printing does not exist, and each formulation should be considered individually.

Another thing to consider is the particle size of constituted powders. Some authors [54], [55] reported that the nozzle diameter should be 50 times greater than the particle size. Others [56], [57] claimed that the ratio between the nozzle diameter and the particle size must be at least 100:1. Otherwise, the nozzle may be clogged, inhibiting the printing process. Since the nozzle orifice for commercial printheads is typically between 22 and 50 μm [58], the particle size should be in the sub-micron range. Moreover, the particles must be well dispersed, forming a stable suspension without agglomerates.

### 2.3 Electric pulse

**Figure 6:**
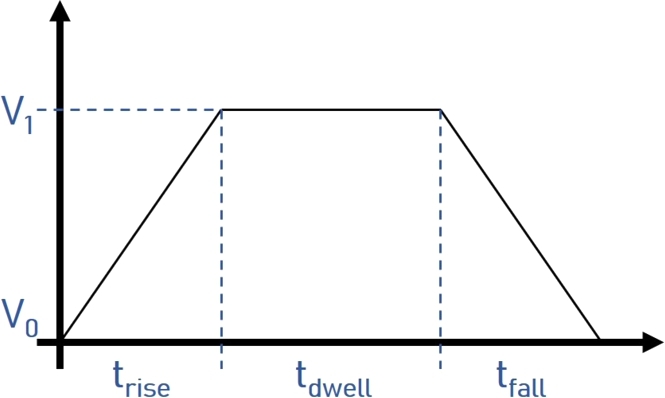
Typical trapezoidal voltage waveform applied to the piezoelectric material in DOD inkjet printing.

The jetting stability, droplet size and velocity are determined by the pulse shape applied to the piezoelectric material [59]. A typical trapezoidal voltage waveform involves a rising time $t_{rise}$, a dwelling time $t_{dwell}$, and a falling time $t_{fall}$ (Fig. 6). The voltage is increased from an initial $V_{0}$ to a final voltage $V_{1}$ during the rise time. Subsequently, the final voltage is kept for the dwelling time, during which the drop is ejected. After that, the voltage is dropped to the initial value $V_{0}$ [60]. This modulation is repeated at a specific frequency (referred to as the jetting frequency) to generate multiple drops during printing.

The waveform design and jetting frequency influence both the ejectability and the shape of produced droplets. Depending on the properties of the ink, these parameters must be adjusted individually. There are no set rules, and they must be optimized experimentally [61], [62].

## 3 Issues of inkjet printing of composite electrodes

### 3.1 Electrode slurry composition

Composite materials applied for the fabrication of electrodes in LIBs should supply the ionic reactants and electrons to the surface of active material (AM) particles. Therefore, they should have both electronic and ${\text{Li}}^{+}$ ionic conductivity [63], [64]. Such a complex ink usually consists of AM, conductive agent (CA), binder, dispersing agent, and solvent. The combination of AM and CA powders should ensure an electrode's mixed ionic and electric conductivity. Binder is necessary to obtain appropriate mechanical properties of deposited films, like toughness, compressibility, and tensile strength. Whereas the stability of produced inks is ensured by a suitable solvent and dispersing agents [65], [66].

AMs used for cathodes are typically layered transition metals oxides (LiMO_2_ (M = Co, Mn, Ni); LiCo_1-x_Ni_x_O_2_; LiNi_x_Co_y_Mn_1-x-y_O_2_), olivines (LiMPO_4_ (M = Fe, Mn, Co, Ni)), or spinels (LiMn_2_O_4_, LiNi_0.5_Mn_1.5_O_4_), all showing upsides and downsides [67]. Anode materials include metal materials (such as aluminum (Al), tin (Sn), magnesium (Mg), silver (Ag), antimony (Sb), and their alloys), conversion-type transition metal compounds (transition-metal sulphides, oxides, phosphides, nitrides, fluorides, and selenides), silicon-based compounds (Si nanoparticles, Si nanotube within a carbon nanotube), and carbon-based compounds (carbon nanostructures, graphene) [68].

**Figure 7:**
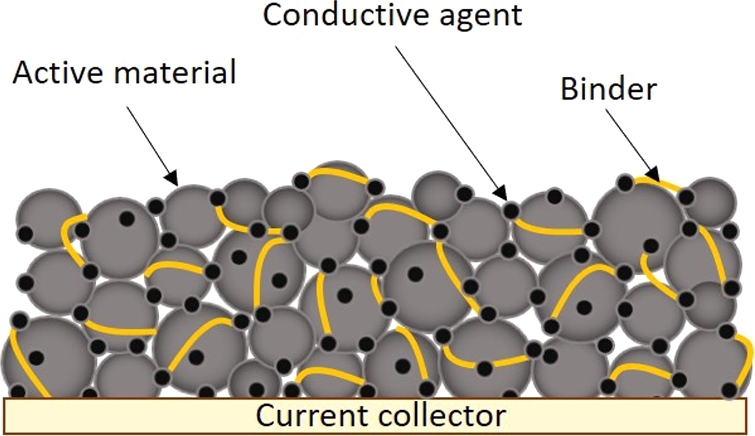
Schematic representation of a typical electrode structure.

The performance of LIBs is greatly influenced by the distribution of components within the electrode (Fig. 7). To achieve the connectivity, AMs and CAs should be evenly distributed with minimal agglomeration and uniformly covered by a binder. Furthermore, strong adhesion between the electrode and the current collector is necessary [69]. Therefore, slurry preparation is crucial in fabricating composite electrodes [70].

This is especially true in inkjet printing technology, where a variety of factors can influence the drop ejection process. The particular nature of ink dictates the parameters of printing. However, ejection is sometimes impossible for various reasons, including rheological characteristics, particle size, and so on. The following sections will go over these points.

### 3.2 Particle size

The particle size of suspended powders is a critical requirement for inkjet printing technology. As mentioned in a previous section, the nozzle diameter limits the maximum particle size. Therefore, nanoparticles should often be implemented in the ink formulation, which may be expensive and dangerous for both health and the environment [71]. Moreover, some powders may not be available in a proper granulometry range; thus, a grinding technique must be employed as a pre-processing step.

Pan et al. [72] reported the degradation of LiNi_0.4_Mn_0.4_Co_0.18_Ti_0.02_O_2_ (NMC) layered structure induced by ball milling, which directly influences the electrochemical properties. Along with the structure damage, the grinding caused a reduction of transition metals and the formation of lithium carbonates, significantly decreasing the Coulombic efficiency and initial capacity. Some authors demonstrated that the application of various coatings on the surface of nickel-rich cathode oxides, for instance, SiO_2_ [73], Al_2_O_3_ [74], ZrO_2_ [75], TiO_2_ [76], AlF_3_ [77], is an effective strategy for increasing the electrochemical performance of batteries [78]. These layers prevent the side reactions between electrode and electrolyte, improving the cycle stability. However, they are usually lithium-ion insulators, negatively impacting the rate capability and discharge capacity of cathodes [79]. Therefore, other materials, which are lithium-ion conductors, were proposed: LiAlO_2_ [80], Li_2_ZrO_3_ [81], LiPON [82], Li_3_PO_4_ [83]. They act as protective layers, ensuring rapid lithium-ion transportation from the cathode material to the electrolyte. Nevertheless, implementing of a grinding step is detrimental to the core-shell structure, and the benefits of the protective coating are lost. Therefore, the powder, which is suitable in terms of printability, may no longer be applicable for electrode formulation.

On the contrary, Fey et al. [84] showed that ball milling of LiFePO_4_ powders might positively affect the electrochemical performance of the battery due to a decrease in particle size without a structure degradation.

Some authors [85], [86] recognized that the reduction in particle size might significantly improve the lithium-ion batteries' performance due to a shorter transport distance for electrons and ions. However, smaller particles yield larger specific surface areas, which results in more side reactions [78]. Jo et al. [87] studied the influence of LiCoO_2_ cathode particle size on the electrochemical performance. They investigated powders of 50 nm, 100 nm, 300 nm, and 1 μm, proving that smaller particles cause an increase in the thickness of the solid electrolyte interphase (SEI) layer (a passivation layer on electrode surfaces originated from decomposition products of electrolytes). As a result, the best rate capacity was observed for 300 nm particles. Aklalouch et al. [88] showed that LiCr_0.2_Ni_0.4_Mn_1.4_O_4_ spinels with particle sizes greater than 500 nm yield better cycling stability than those with particle sizes smaller than 500 nm. Yang et al. [89] reported the optimal particle size of LiMn_0.8_Fe_0.2_PO_4_ powder as cathode material for Li-ion batteries with improved electrochemical performance. They proved that the electrode with the particle size of ∼9.39 μm may reach higher initial discharge capacity and higher capacity retention, compared to those, with the particle size of ∼2.71 μm, ∼3.74 μm, ∼6.41 μm or ∼16.31 μm. Bläubaum et al. [90] studied the influence of particle size of spherical graphite as an anode material on the performance of lithium-ion batteries. The results demonstrated that smaller particles and a narrow distribution yield better cell performance than coarser and broader distributions. However, there is a threshold below which the decrease in particle size negatively affects electrode properties. Due to strong SEI growth, smaller particle sizes result in a decrease in capacity. In that article, it was proven that this deleterious effect could be observed for particle sizes smaller than 1.5 μm.

Apart from the particle size, the particle morphology is also of great importance for the electrochemical performance of LIBs, as it is directly linked to the electrode's effective surface area [91]. Prior research suggests that the spherical shape of particles may be beneficial. It offers higher tap density, which influences the energy density of batteries. Furthermore, the surface of the active materials is uniformly covered with a conductive agent, which improves performance [92], [93]. It was observed that spherical particles lead to a longer life cycle compared to irregular particles with rough surfaces. Since the specific surface area of the latter is higher, the relative volume changes during the lithiation/delithiation process are more prominent, reducing the capacity retention [94].

Regarding the IJP technique, the biggest possible particle size is dictated by the nozzle diameter, which is a strict requirement. However, there is an optimal particle size for electrode performance, which often may be greater than the limit for the drop ejection. After grinding, which is frequently required for the process, the spherical particle shape, regarded as advantageous for lithium-ion battery fabrication, is lost. Hence, to use the IJP method, sometimes the most favorable particle properties must be banished in the interest of printability, which results in a compromise. Yet, it must be understood that the objective is not simply to print an electrode, but to print a particular electrode with greater performance than one deposited by conventional fabrication methods. As it was evidenced in the literature, powder milling can significantly deteriorate material structure, resulting in poor electrochemical characteristics. Moreover, larger particle sizes are sometimes beneficial in terms of final product functionality. Thought must therefore be given to the practicality of the IJP application, taking into account the final product performance. Before deciding to use the IJP, the effect of particle size and shape on electrochemical characteristics should be evaluated. While reaching the STOP points on the flowchart (Fig. 2), it may be more reasonable to use alternate manufacturing methods that do not have such strict particle size constraints.

### 3.3 Viscoelasticity

Although the polymeric binder does not contribute directly to the electrochemical benefits of batteries, it significantly impacts their performance [95], [96]. Its principal function is interconnecting the components (active material, conductive agent) and ensuring their adhesion with a current collector during the lithiation/delithiation process. When there is a lack of effective physical contact between the particles, the electrical conductivity and the electrochemical performance of the electrode are dramatically decreased [97], [98].

For several years, great effort has been devoted to studying binders with application for battery fabrication. The majority of prior research focused on their influence on the performance of electrodes, investigating binding mechanism [99], [100], polymer architecture [101], electrochemical performance [102], [103], electrochemical stability, mechanical properties [103], [104], ionic conductivity [105], [106], etc.

Many polymers were reported as potential candidates for a binder in lithium-ion battery components. For instance, PVDF [107], [108], sodium carboxymethyl cellulose (CMC) [109], poly(acrylic acid) (PAA) [110], polyaniline (PANI) [111], PEDOT:PSS [112], sodium alginate (Alg) [113], *β*-cyclodextrin (*β*-CDp) [114] were presented, as prominent candidates for silicon-based anodes. Among them, CMC is one of the most common candidates [115], considerably improving the cycling stability of electrodes [116], [117]. PVDF [118], PAA [119], nafion [120], polyimide [121], cellulose [122] are the examples of effective binders for layered transition metal oxides cathodes. Polymers used for solid electrolytes must be characterized by high ionic conductivity, ensuring a proper ${\text{Li}}^{+}$ ion diffusion [96]. Previous research suggests numerous promising materials, such as polyethylene oxide (PEO) [123], polyacrylonitrile (PAN) [124], polytetrafluoroethylene (PTFE) [125], cellulose [126], and nafion [106], of which the former is dominant. A detailed discussion of host materials for ${\text{Li}}^{+}$ – conducting solid polymer electrolytes may be found in the interesting work of Mindemark et al. (see [127]).

Although numerous studies have investigated the impact of polymeric binders on battery performance, the research on the viscoelastic behavior of the polymeric inks and their processability is limited. For conventional manufacturing methods, for instance, tape casting, it is of low importance. However, using the IJP process, an unsuitable polymer can hinder the drop ejection, making the deposition impossible.

In the previous part of the article, the fluid rheological requirements for a printable ink were presented. However, the Z value, which determines the window of printability relating to the surface tension, viscosity, and density of the ink, does not consider non-Newtonian properties, such as shear-thinning or viscoelasticity. The presence of polymer in the ink has a significant impact on the drop generation and ejection process [128]. Additional dimensionless groups can be used to describe viscoelastic fluid behavior, but this is beyond the scope of this review, and interested readers should refer to the paper of Clasen et al. [129]. We want to highlight the difficulties in determining the operational window for functional inks with the presence of binders. The authors classified the dimensionless groups into two categories: the material-property-based, which determines the main mechanism dominating the initial fluid breakup, and the dynamic groups defining the primary mechanism during the shear thinning behavior. The dominant mechanism may change during jetting. As Li and Deng [130] long since stated, the influence of non-Newtonian fluids on the dispensing process needs to be better understood.

**Figure 8:**
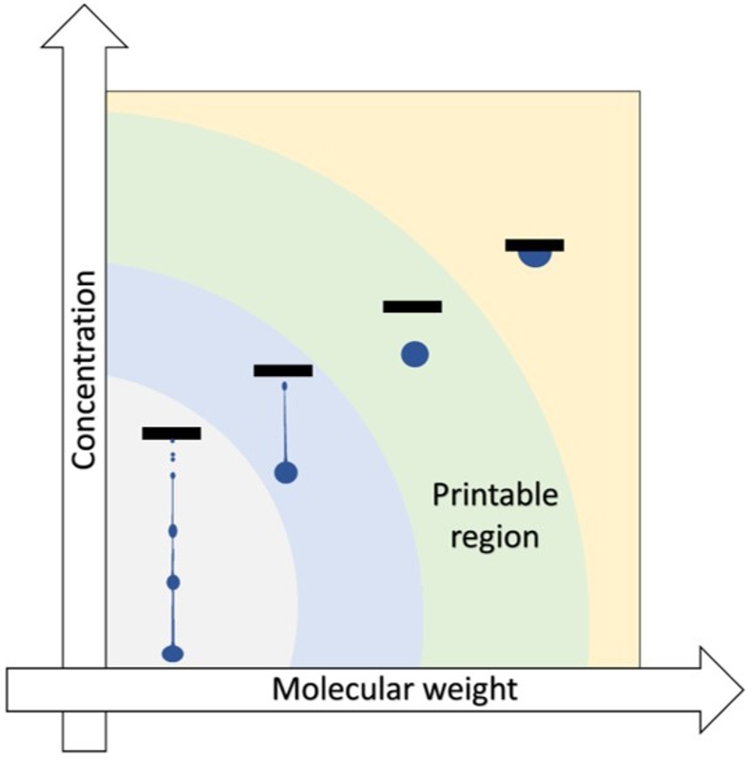
Four regimes in inkjet drop generation as a function of concentration and molecular weight of polymer.

Some authors have proposed distinct scenarios (Fig. 8) in inkjet drop generation depending on the combination of molecular weight and concentration of polymer used [128], [131]. At very low molecular mass/concentration, a long ligament is created, which simultaneously breaks up, creating the satellite droplets. Within this regime, the print quality is very poor. When raising the molecular mass/concentration, fewer satellites are created, making the ejection less chaotic. Increasing the concentration or molecular weight even more yields the optimum print quality, ejecting a single drop without a ligament. The last regime determines the limit of printability. At high concentration/molecular weight, the behavior of polymeric ink is dominated by a viscoelastic behavior, hindering the drop ejection. The high viscosity of inks containing high molecular weight polymers at high concentrations is not the only impediment to their jetting. Even inks formulated with such polymers, but with relatively low viscosity (values adapted to the IJP, i.e., up to 20 mPa.s), may experience jetting problems.

**Figure 9:**
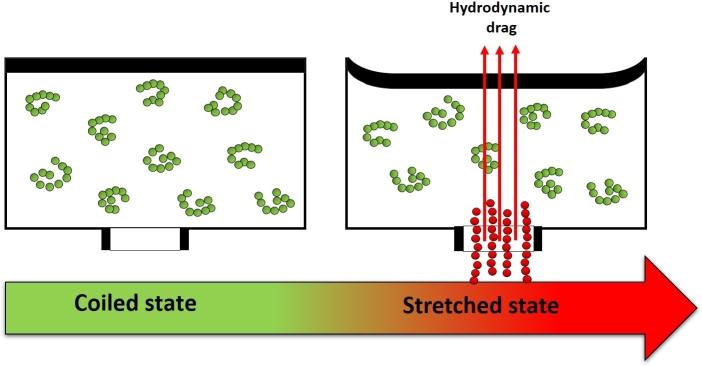
Schematic representation of strain hardening phenomenon: a) coiled state of polymeric chains, when no stress is applied b) stretched state of polymeric chains during the ejection.

A possible explanation for this may be the strain hardening, which plays a key role when determining ink printability. Fig. 9 schematically represents this phenomenon. If no stress is applied, polymeric chains inside the cartridge are coiled. During the ejection, a piezoelectric element is deformed, and a high strain rate is generated at the nozzle exit, causing the alignment of polymeric chains – transition to a stretched state. This results in a strong increase in the hydrodynamic drag force, which impedes fluid flow [128], [132].

The fluids in piezoelectric inkjet printheads are exposed to frequencies of 10–100 kHz [133], which are likely to cause the coil-stretch transition. However, there are difficulties in the rheological characterization of inks under these conditions. The highest accessible frequencies in commercial rheometers are limited. Usually, they are a few orders of magnitude lower than those applied in inkjet printers. Simple metrics, such as low shear rate viscosity, are insufficient to evaluate jetting behavior. Therefore, it is generally observed during the ejection via direct imaging. A camera takes a series of pictures of the drop generation, enabling the evaluation of ink printability and print quality [129]. Nevertheless, the fundamental explanation for fluid behavior cannot be provided.

Hoath et al. [134] reported the DOD jetting behavior of weakly elastic linear polystyrene (PS) diluted in diethyl phthalate (DEP) with different molecular weights. They proved that the increase in the molecular weight of the polymer causes a delay in the break-off time and a decrease in drop speed, which is probably caused by the polymer molecules stretching in the filament. Gans et al. [135] showed that the printability of polydisperse polystyrene in acetophenone decreases with molecular weight and concentration of polymer, which is caused by the elastic stresses arising from elongational flow in the nozzle. McIlroy et al. [136] developed a model to predict the jetting behavior of diluted polymer solution in DOD inkjet printing. They assumed that the maximum polymer concentration, which can be jetted, depends on the molecular weight and the solvent used. Another important factor is nozzle geometry, which can significantly impact printability. Molecules may also become damaged under the high strain rates at the nozzle exit, losing their initial properties. Xu et al. [128] investigated the effect of cellulose ester (CE) polymers' concentration on the drop formation during the DOD IJP by visual observations of a ligament. They assumed that the polymeric inks formulated below the coil overlap concentration show very similar behavior regardless of the molecular mass. Above this point, the elastic nature of the ligament dramatically increases, as a result of chain entanglement, and in extreme cases, may prevent ejection. Additionally, cellulose esters, as weakly associating polymers, can form a gel structure by breaking H-bonds and reforming them in their extended form. This structure acts as physical crosslinks, and its viscosity may reach high values. Yoo et al. [137] studied the drop generation experimentally on xanthan gum solutions in water-glycerin mixtures. They assumed that the drop generation process could be divided into two main mechanisms: ejection, which is driven by a high shear viscosity, and detachment, which is controlled by extensional viscosity. As a consequence of the strain hardening phenomenon, the extensional viscosity becomes very large, retarding the extension of a ligament. Mun et al. [138] examined dilute and semidilute poly(ethylene oxide) solutions' behavior while jetting through a nozzle, proving that the breakup depends on both the polymer molecular weight and concentration. A-Alamry et al. [139] reported the mechanical degradation of polymer molecular weight induced by the flow during DOD inkjet printing for PMMA and PS. It was shown that the degradation does not occur for molecular weights smaller than 100 kDa, nor for those larger than 1000 kDa. The polymer deterioration is more pronounced between these values at high elongation rates and with higher polydispersity polymers. Gans et al. [132] studied the impact of the polymer architecture on drop generation during inkjet printing. They compared linear and 6-arm star PMMA, showing that the latter, with less pronounced filament formation, is more suitable for this technology, and can be a beneficial additive in the ink's formulation. However, the possible polymer degradation was not examined. Aqueous solutions of PEDOT:PSS [140], [141] were presented as excellent inks for inkjet printing technology. Within the nozzle, they exhibit low viscosity, making the ejection possible, and once the drop is formed, they recover a high viscosity, creating satellite-free deposition. The difficulties in ejection were not reported, which may indicate outstanding printability of this polymeric solution.

Based on the examples, a universal window of a stable drop generation for polymeric solutions may not exist. Furthermore, although many authors studied the influence of the polymer concentration and molecular weight on ink printability, many other factors may be influential, for instance the nature of the polymer, its architecture, the kind of solvent, etc. Therefore, there are too many variables to put forward a universal framework that would be valid for all polymeric solutions.

A major challenge is the preparation of the slurry, which would be suitable in terms of the rheological properties and battery performance. Gordon et al. [142] investigated the impact of CMC concentration, molecular mass, and degree of substitution on the electrode's electrical conductivity and mechanical integrity. The influence on flow behavior was also presented, but only the basic rheological measurements, which are not sufficient to evaluate printability. They concluded that the electrical conductivity decreases with an increase in binder concentration, and decreases when CMC with higher molecular mass is employed. The degree of substitution does not have a significant impact. As for the mechanical strength, it is significantly improved when the CMC concentration increases.

Therefore, it can be seen that it is very challenging to adapt the polymeric ink properties for both the final electrode performance and the processability. It causes difficulties, especially in inkjet printing, where the ink requirements are very strict. Thus, there is often a trade-off between the battery efficiency and the suitable rheological properties of electrode slurries. Although many authors have conducted studies, this problem still needs to be explored. Only a few works in literature (see Table 1) demonstrate the printability of complex inks for the application of batteries, which in addition to a polymer and a solvent, contain diverse powders and additives such as dispersing agents. However, some results show that the ejection of a drop is impractical, but the explanation is missing [143]. It should be pointed out that for industrial applications and research, functional polymeric inks are much more frequently applied than Newtonian fluids, and predicting their printability is highly significant. Therefore, a more systematic and theoretical analysis is required for the interaction between each component of the inks and their flow behavior.

**Table 1:** Components of LIBs fabricated by the IJP method.

Active material		Solvent	Additives	Printer	Printed layer thickness	Electrochemical performance of printed structure	Ref.
Cathode	LiCoO_2_	DI-water	CMC, commercial surfactant solution	Canon BJC-1000sp	1.2 μm (30 layers)	120 mAh/g at 180 μA/cm^2^, 95% capacity retention after 100 cycles	[30]
	LiFePO_4_	buffer solution (HCl + NaOH)	CMC, TritonX-100, glycerin	Dimatix-2800	20 μm	129.9 mAh/g at 0.1 C (Al foil), 151.3 mAh/g at 0.1 C (CNT)	[164]
	LiFePO_4_	DI-water	PAMA	piezoelectric ink-jet printer	4 μm (40 layers)	80 mAh/g at 9 C, 70 mAh/g at 90 C	[143]
	LMNCO	NMP	PVDF, surfactant	Dimatix-2831	11.5 μm (25 layers)	240 mAh/g at 0.01 C	[166]
	1.20 NCM/1.25 NCM	NMP	PVDF, ethylene glycol, diethylene glycol, propylene glycol	Dimatix-2831	–	–	[145]
	V_2_O_5_/MXene	–	–	Dimatix-2800	–	321 mAh/g at 1 C, 91.8% capacity retention after 680 cycles	[169]
Anode	SnO_2_	DI-water/absolute ethanol/diethylene glycol/triethanolamine/isopropylalcohol	CMC, CH10B, CH12B	Canon BJC-1000sp	2.3 μm (10 layers)	812.7 mAh/g at 33 μA/cm^2^	[16]
	Li_4_Ti_5_O_12_	aqueous solution (LDS+Li-PAA)	PVP	Flat-bed Breva thermal inkjet	3.3 μm (20 layers)	128 mAh/g at 0.5 C	[168]
	Si	DI-water	PEDOT:PSS, PVP, CMC, sodium alginate	HP Deskjet 2540	1 μm (25 layers)	>1700 mAh/g for 100 cycles, capacity retention of over 1000 cycles at 1000 mAh/g	[112]
	Graphene	ethanol and terpineol	–	Jetlab-4	268 nm (8 layers)	942 mAh/g at 0.1 C, 87% capacity retention after 100 cycles	[167]
	TiO_2_	DI-water	PEDOT:PSS/PVP/PVDF	HP Deskjet 2540	3.02 μm (25 layers)	180 mAh/g at 0.1 C	[146]
	Li_4_Ti_5_O_12_	DI-water/absolute ethanol/diethylene glycol/	CMC, CH10B, CH12B	Canon 1000SP	1.7–1.8 μm (10 layers)	174 mAh/g at 10.4 μA/cm^2^, 88% capacity retention after 300 cycles	[17]
Electrolyte	Ionogel	–	–	Dimatix-2800	5 μm (4 layers)	60 mAh/g at 0.1 C for LiFePO_4_/ionogel/Li_4_Ti_5_O_12_ full cell	[144]

Abbreviations utilized in the table: CMC – carboxymethyl cellulose; CNT – carbon nanotube paper; DI-water – deionized water; LDS – lithium dodecyl sulfate; Li-PAA – lithium polyacrylate; LMNCO – Li_1.15_K_0.05_Mn_0.54_Ni_0.13_Co_0.13_O_2_; 1.20 NCM – Li_1.2_Mn_0.54_Ni_0.13_Co_0.13_O_2_; 1.25 NCM – Li_1.25_Mn_0.54_Ni_0.13_Co_0.13_O_2_; NMP – N-methyl pyrrolidinone; PAMA – poly-acrylic-co-maleic acid; PEDOT:PSS – poly(3,4-ethylenedioxythiophene) polystyrene sulfonate; PVDF – polyvinylidene fluoride; PVP – polyvinylpyrrolidone.

### 3.4 Rheological characteristics

Surface tension and viscosity are the first parameters to be adjusted to allow drop generation. Printer manufacturers set their values which usually fall within a narrow range. For instance, Fujifilm recommends a viscosity of 10–12 mPa.s, and surface tension of 28–33 mN/m for Dimatix 2800 [144] and 8–10 mPa.s, 28–32 mN/m for Dimatix 2831 [145]. Lawes et al. [112], [146] used HP Deskjet 2540 inkjet printer to fabricate Si and TiO_2_ composite electrodes. They adjusted ink viscosity to the value of 10 mPa.s by an appropriate volume of deionized (DI) water. However, information concerning surface tension is missing.

It can be difficult to tailor both viscosity and surface tension simultaneously. The choice of a solvent greatly influences the rheology of a suspension. However, many other factors, such as environmental policy, accessibility, cost, technological requirements, etc., constrain it. First and foremost, the solvent should be directly aligned with the binder. The binder must be dissolved, and its architecture (which may differ depending on the solvent nature) should be beneficial for the final product performance. For instance, the structure of PEDOT:PSS alters from a continuous network to separated clusters with an increase in pH, which affects the conductivity [147]. Most of the time, after selecting a proper solvent, additives must be added to adjust the ink's rheological properties. Co-solvents and surfactants are useful additives, but care must be taken when choosing them because chemical or interparticle interactions can occur, destabilizing the formulation. Moreover, the impact of the additives on the electrode performance must be evaluated. Finally, the environmental benefits of new-generation LIBs or ASSLIBs should be explored [148]. The replacement of N-methylpyrrolidone (NMP) used in traditional processing by aqueous suspensions eliminates the serious risk of toxicity for humans. Moreover, it facilitates battery recycling by integrating water-soluble binders into the electrode, significantly simplifying the conversion of solid electrodes to aqueous black matter [149].

All in all, from the processing point of view, the solvent and additives must be adapted according to other constituted ink components and required rheological characteristics. Furthermore, on the functionality side, the impact of the formulation on the electrochemical performance must be evaluated. Finally, from the environmental perspective, ink processing should provide benefits, making modern LIBs less toxic and environmentally friendly. Therefore, to advance in the field, each of these points and their mutual influence must be examined, and more importance must be placed on ink formulation.

### 3.5 Dispersion/stability

Previously, this paper discussed the importance of particle size in slurry preparation for the IJP. Particles that are too large could clog the nozzle, and also agglomeration of them can impede the printing. Therefore, functional inks should be sufficiently well dispersed and stable.

Since powders for the IJP application are in the submicrometer or nanometer size range, the behavior of particles in a slurry is governed by surface chemistry. According to the well-known DLVO theory [150], the stability of aqueous colloidal dispersions depends largely on interparticle forces, among which van der Waals attraction and electrostatic repulsion play a major role. Balancing them, a well-dispersed system may be obtained, where every particle is separated from one another. The colloidal stability of simple suspensions (one kind of powder without additives) can be achieved when electrostatic repulsion, linked to the electric double layer (EDL), is dominant and counteract the attractive van der Waals forces. However, in complex suspensions, stability can also be affected by other forces, for example, steric repulsions. Therefore, the stability of complex aqueous suspensions may be enhanced by controlling the electrostatic repulsion (for example, adjusting the zeta potential by a variation of pH) and modifying the surface chemistry, or by using additives such as dispersing agents [151]. In nonaqueous systems, the EDL mechanism is a complex topic, and electrostatic interactions between particles are usually of minor importance. Accordingly, steric stabilization is dominant in nonaqueous colloids [152], [153].

Previous studies have shown that slurry preparation may significantly influence the suspension microstructure, and consequently the electrochemical performance of electrodes. Zhang et al. [154] demonstrated better discharge capacity and cycling stability of LiFePO_4_ cathode with the addition of Triton-100 as a dispersing agent, which is directly linked to a more uniform dispersion of powders. Zhang et al. [155] used Triton X-100 for the pre-dispersion of carbon black in LiFePO_4_ cathode slurry, improving the electrochemical performance of the electrode. Porcher et al. [156] tested three kinds of dispersing agents: an anionic one, a non-ionic one, and a cationic one, studying their impact on the LiFePO_4_ electrode slurry properties, as well as on the microstructure and electrochemical performance of the tape casted composite cathodes. Ionic surfactants were reported to be unsuitable due to the corrosion of the aluminum current collector. Optimal electrochemical performances, directly related to the microstructure, were obtained with a non-ionic surfactant. Its concentration also has a significant impact, and was adjusted so that the dispersant fully covers the carbon black (CB) surface. Sometimes, more than one dispersing agent might be adapted to ensure that an effective product is tailored for each of the constituted powders [16], [17].

Not only do dispersing agents impact the dispersion state of a slurry, but also binders. Depending on the composition of the ink, its presence may be favorable or disadvantageous [65], [157]. In the work of Lawes [146], it was shown that TiO_2_ inks prepared with polyvinylpyrrolidone (PVP) and polyvinylidene fluoride (PVDF) as binders are agglomerated and clog the nozzle of the ink-jet printer. However, using poly(3,4-ethylenedioxythiophene)-poly(styrene sulfonate) (PEDOT:PSS), he obtained a homogenous and printable suspension.

Another possible way to obtain well-dispersed slurries is by modifying the particle surface in order to create functional groups. It is known that carbon materials, used as a conductive agent for electrodes, have hydrophobic properties and strong van der Waals forces resulting in self-aggregation in aqueous mediums. Therefore, in order to increase their hydrophilicity and dispersibility, chemical treatments can be applied. However, it was reported that this strategy unfavorably influences their electronic conductivity [158], [159].

Apart from choosing a suitable dispersing agent, the slurry preparation process considerably influences particle distribution. Kim et al. [70] prepared several LiCoO_2_ cathode formulations, varying the mixing sequences and investigating their impact on the dispersion states of the powders. It was shown that the mixing sequence affects the microstructure of suspension, and accordingly the performance of batteries. Konda et al. [69] demonstrated that employing the dry ball-milling as a pre-mixing step can result in enhanced electrochemical characteristics of LiFePO_4_ electrodes. Lee et al. [160] compared two different mixing processes and their impact on the LiCoO_2_ cathode performance. It was shown that more uniform powder distribution is obtained by adding a solvent into a solid mixture stepwise instead of introducing the total amount at once, resulting in better electrochemical properties. Akuzum et al. [161] studied the effect of mixing techniques (stir-bar and high-speed shear mixing) and dispersion time on the carbon electrodes' performance. They proved that minimal changes in slurry preparation protocol may result in huge differences in electrochemical performance. Capacity differences up to 90% were achieved by optimizing mixing time and methods. Waluś et al. [162] employed a high-energy mechanical dispersion (Dispermat) for sulfur electrode ink preparation, showing its positive impact on the electrochemical behavior of the battery.

Hence, it is clear that slurry preparation is of great importance for electrode performance. Nevertheless, unlike the conventional fabrication processes, for instance tape casting, in the case of the IJP method, the dispersion influences not only the final product's properties but also the printability. Very often, small particle agglomeration may be detrimental to printing; therefore much more attention must be addressed to this issue. Moreover, the impact of the additives on the final product's performance must be evaluated. Sometimes a large amount of a dispersing agent is required to obtain a stable ink [163], and in such cases a more thorough study is needed.

## 4 Current achievements by inkjet printing

Despite the many advantages of the IJP method in manufacturing LIBs, only a few studies have been done on the subject. Table 1 summarizes the publications, highlighting the most pertinent information.

The first reports on the application of the IJP to lithium-ion batteries were published by Shanghai Key Laboratory of Molecular Catalysis and Innovative Materials in 2006 [16], 2008 [30] and 2009 [17]. They described the preparation process of SnO_2_, Li_4_Ti_5_O_12_ and LiCoO_2_ inks, proposing ball milling and/or ultrasonic bath for homogenizing, all done in the correct sequence. Printing was successfully performed with the Canon BC-03 cartridge, but the authors did not provide any information concerning the ink requirements and properties (viscosity, surface tension, stability, etc.).

In 2015, Gu et al. [164] used the IJP process to fabricate LiFePO_4_ cathode thin films. The ink was prepared by bath sonication, and prior to printing, it was centrifuged to eliminate the possible large particles. The viscosity was set to 13 mPa.s, the recommended setting for the Dimatix-2800 printer. The electrochemical performance of inkjet-printed cathode layers was compared to those deposited by the tape casting method, with higher capacities obtained for the latter. However, since the formulation of a paste for doctor blading was different compared to the ink, the results should not be indicative.

The same year, Dellanoy et al. presented the possibilities of printing LiFePO_4_ cathode [143] and a silica-based ionogel electrolyte [144]. For the cathode fabrication, they used a piezoelectric inkjet printer specifically designed for R&D with the following ink requirements: viscosity 10–12 mPa.s, surface tension 28–33 mN/m, particle size <200nm. Three formulations with different additives were tested: with PAMA, with CMC, with CMC + Triton X-100, prepared by magnetic stirring and ball milling. Basic rheological measurements were performed, verifying viscosity and stability at rest (storage modulus and shear modulus). Printability tests revealed that inks containing CMC could not be ejected. The ink with PAMA was printable, possibly due to its low viscosity at the high shear rate and high stability. However, it is a probable assumption, and a more thorough examination is needed to understand the link between printability and rheological properties. Furthermore, PAMA has a lower molecular mass than CMC, which may significantly impact ink processability. The inkjet printed electrode exhibits excellent cyclability and very high rate charge/discharge behavior. For the printing of the electrolyte, Dimatix DMP2800 ink-jet printer was used. The viscosity of ionogel was measured, however other ink characteristics are not presented. The inkjet printed electrolyte was tested with a full lithium-ion cell, demonstrating competitive performance compared to those based on expensive PVD processes.

Lawes used HP Deskjet 2540 inkjet printer for the deposition of TiO_2_ [112] and Si [146] anodes. For both materials ink was prepared by sonication, and viscosity was adjusted to 10 mPa.s, which the printer manufacturer recommends. Three kinds of binders were tested for TiO_2_ electrode formulation: PVDF, PVP, and PEDOT:PSS, and only the latter was printable. Slurries prepared with PVDF and PVP were agglomerated and clogged the nozzle. PEDOT:PSS, PVP, CMC, and Na-alginate were used as binders for the preparation of Si anode. It was reported that each ink was sufficiently well dispersed and printable. However, anodes with PEDOT:PSS showed the highest stability due to their electrical conductivity and reversible deformation during electrode cycling.

Maximov et al. [145] fabricated NMC cathode using the IJP. Ink was dispersed by an ultrasonic bath, and large agglomerates were eliminated by centrifugation. They used NMP, the most common solvent for cathode fabrication in the battery industry. However, due to its toxicity, it is restricted in several countries [165]. As for the rheological properties of prepared inks, authors adjusted the viscosity and surface tension according to the printer's requirements, i.e., 8–10 mPa.s, and 28–32 mN/m, respectively. They studied the influence of different additives (ethylene glycol, diethylene glycol, propylene glycol) on the slurry characteristics. The stability was evaluated by the *ζ*-potential measurement. Although the authors determined the optimal printing conditions, they did not present any results for the inkjet printed layers.

Kolchanov et al. [166] formulated LMNC-based cathode inks, optimizing the rheological properties for the IJP: viscosity, surface tension, and contact angle. The Z value was calculated based on the measurements, giving an idea about printability. The concentration of dispersing agent was adjusted, and the sedimental stability was studied using centrifugation. Printability tests revealed that a stable drop might be generated, and a cathode thin film was fabricated. Its electrochemical performance was compared with the electrode deposited by the tape casting method, resulting in similar values.

Kushwaha et al. [167] showed the possibility of inkjet printing of a graphene film as an anode for lithium-ion batteries. In the article, it is mentioned that the stability and viscosity of ink are critical for printability, but studies on the rheological properties are not comprehensive enough.

Viviani et al. [168] investigated the effect of carbon additives on the electrochemical performance of inkjet-printed Li_4_Ti_5_O_12_ anodes, fabricating them using a thermal inkjet printer. The rheological measurements were performed to confirm the suitability of slurries for the process. Although the Z value was greater than 10, inks were printable and anodes were successfully deposited. Furthermore, the electrodes manufactured with the carbon nanotubes (CNTs) as a conductive agent showed the highest specific capacity among all printed films, demonstrating that CNTs improving electrochemical performances may be applied to the IJP method.

Wang et al. [169] fabricated V_2_O_5_/MXene heterostructure cathode for lithium-ion batteries using the IJP. The prepared inks were printable, but the authors did not provide information concerning their properties. Nevertheless, the printed cathode layers exhibited excellent electrochemical performance, offering new possibilities for two-dimensional heterostructures in high-performance batteries.

Although the rheological properties of slurries are crucial in determining the printability of inks, the previous studies present either only basic rheological measurements, or the characteristics of the fluids are completely omitted. Furthermore, inks used to fabricate LIBs are very complex, and their flow behavior greatly impacts processability. Therefore, a more in-depth examination is needed to understand the relationship between rheology and printability. Moreover, the work done on the topic focuses exclusively on the printing of thin films, and as far as we know, no previous research investigated the inkjet printing of 3D structures for LIBs.

## 5 Concluding remarks and future scope

Implementing inkjet printing technology may be a prospective development path in the field of lithium-ion batteries. Not only can novel three-dimensional electrodes with high accuracy be created, but also thin-film electrodes, which often yield greater electrochemical performance than those deposited by conventional tape casting techniques. However, formulating a suitable ink, which may be successfully deposited using the IJP method, is challenging. The properties of a slurry impact not only the final product's performance, but also the printability. A suspension that is not properly tailored can impede the ejection, making the process impossible.

The first difficulty to overcome is the adjustment of the rheological properties of the ink in accordance with the requirements of printer manufacturers. In general, the values of surface tension and viscosity are dictated by these requirements. These two parameters chart the window of printability, which is often narrow, and simultaneously adapting both variables is not easy. Although additives may be helpful, they can also destabilize the suspension, cause unwanted reactions, or negatively impact the electrochemical performance of the final product.

Another point to deal with is the particle size of powders constituting the ink. The nozzle diameter dictates the largest possible size and it should be strictly respected. Otherwise, the printing may be impossible due to the nozzle clogging. However, very often the electrochemical performance of batteries can be greatly improved by modifying particle size and morphology. The smaller size is only sometimes the optimal one. Furthermore, the spherical shape reported as beneficial for battery performance no longer exists after the grinding step, which is frequently required.

Obviously, particles that are too large may clog the nozzle, but also agglomerates can impede the printing. Therefore, inks should be well dispersed, which is not only mandatory for printability, but also favorable for electrode performance. The dispersion state of slurry is of great importance regardless of the fabrication method, but in the case of the IJP, much more attention should be given to the issue.

Another key problem, which is very often omitted in the literature, is the impact of polymer on the suspension properties and printability. The slurry characteristics may be dramatically changed by adding a binder, an essential element in functional inks for battery fabrication. Initially, the strain hardening problem must be overcome to eject a droplet, which can be done by modification/replacement of polymer. Furthermore, since the functional inks often exhibit non-Newtonian or viscoelastic behavior, it may be problematic to anticipate the drop generation process. A variety of factors influence printability, and the process parameters should be adjusted individually for each particular ink.

To sum up, a limited number of studies have been devoted to the fabrication of LIBs by the IJP technique. The small amount of research can be attributed to the challenge of formulating suitable electrode inks while maintaining good electrochemical properties. Until now, the studies have focused mainly on the ability to print thin films, with little attention given to the rheological properties of slurries. This review highlights the importance of thorough characterization of electrode inks. In order to advance in the field, a more in-depth examination is needed to understand the relationship between rheology and printability.

## Declarations

### Author contribution statement

All authors listed have significantly contributed to the development and the writing of this article.

### Funding statement

This work was supported by 10.13039/100010671H2020 LEIT Advanced Materials (875029).

### Data availability statement

No data was used for the research described in the article.

### Declaration of interests statement

The authors declare no competing interests.

### Additional information

No additional information is available for this paper.
